# Catheter related venous thrombosis with cooling and warming catheters: two case reports

**DOI:** 10.4076/1757-1626-2-8857

**Published:** 2009-09-08

**Authors:** Bertrand Prunet, Guillaume Lacroix, Julien Bordes, Raphael Poyet, Erwan D'Aranda, Philippe Goutorbe

**Affiliations:** 1 Intensive Care Unit, Sainte Anne Hospital, Boulevard Sainte Anne, 83000 Toulon, France; 2 Department of Cardiology, Sainte Anne Hospital, Boulevard Sainte Anne, 83000 Toulon, France

## Abstract

**Introduction:**

Intravascular cooling and warming catheters are among a range of proliferating technologies used for temperature control. Complications related to the use of these devices are few, and no definitive evidence has been presented thus far to indicate any differences in complication rates between these balloon catheters and other central vein catheters. We report two cases of cooling and warming catheter-related venous thrombosis. They are the both first ones report of this kind in the literature.

**Case presentation:**

The first case was a 17-year-old man admitted with severe head trauma. On day 6, he presented with severe intracranial hypertension, requiring increased medical treatment: mannitol osmotherapy, barbiturate-induced coma, and mild therapeutic hypothermia. A double-lumen Alsius CoolLine catheter was placed in the inferior veina cava via the left femoral vein and active cooling was begun. On day 10, physical examination of the left inguinal area and echo-doppler revealed catheter-related thrombophlebitis with left iliocaval vein occlusion. The second case was a 42-year-old man admitted with a severe burn. On day 2, the patient was taken to the operating room for the first staged excision of his burn wounds. A triple lumen Alsius Icy catheter was placed in the inferior vena cava via the right femoral vein and active core warming of the patient was begun. From day 2 to day 7, active core warming of the patient was maintained. On day 7, he presented with a septic thrombophlebitis. Echo-doppler revealed a 4-cm-long thrombus at the femoral catheter site with complete blood flow obstruction and blood cultures and catheter tip were positive for methicillin-resistant S*taphylococcus aureus*.

**Conclusion:**

Although generally considered safe, cooling and warming catheters can be associated with mechanical complications such as catheter-related venous thrombosis. Intensivists who use these devices should be aware of this possible complication. Finally, as with any other invasive catheter, to reduce the risk of complications, the catheter should be removed promptly when no longer needed.

## Introduction

Intravascular cooling and warming catheters are among a range of proliferating technologies used for temperature control. Their main indication is for the induction of mild therapeutic hypothermia to prevent and/or mitigate various forms of neurologic injury, notably postanoxic encephalopathy following cardiac arrest [1]. Common secondary indications include other situations in which temperature management is warranted, such as fever reduction in the neurologic intensive care unit [1], core warming of burned patients during surgical treatment of large burn injuries [2], and active re-warming for hypothermia associated with traumatic injury [3]. The CoolGard 3000 system (Alsius Corporation, Irvine, CA, USA) uses indwelling central venous catheters (CoolLine or Icy catheters) that can be placed in the femoral vein (catheter with three or two cooling balloons) or the subclavian or jugular vein (catheter with two cooling balloons). For direct cooling of the blood, sterile saline is refrigerated in the external device (CoolGard 3000) and then pumped through the balloons coaxially mounted on the catheter. The catheter contains a temperature probe, enabling a closed-loop temperature control system. The temperature is set to the desired level (range 28-38°C), and the device cools the patient to this level by decreasing or increasing the temperature of the circulating saline. Insertion of central venous catheters carries several inherent risks, including placement errors, bleeding, infection, catheter-related venous thrombosis, pneumothorax, and injury to nearby structures. Complications related to the use of the CoolGard system are few, and no definitive evidence has been presented thus far to indicate any differences in complication rates between these balloon catheters and other central vein catheters [3]. Here, we report two cases of cooling and warming catheter-related venous thrombosis.

## Case presentation

### Case report 1

A 17-year-old French Caucasian man was admitted to our hospital with severe head trauma after a scooter crash. Initial intracranial pressure was normal. On day 6, he presented with severe intracranial hypertension, requiring increased medical treatment: mannitol osmotherapy, barbiturate-induced coma, and mild therapeutic hypothermia. A double-lumen Alsius CoolLine catheter (outer diameter at insertion site: 9.3 F, length: 22 cm) was placed in the inferior vena cava via the left femoral vein and active cooling was begun. On day 7, the patient was taken to the operating room for an external ventricular derivation and decompressive craniotomy. On day 10, physical examination of the left inguinal area revealed thrombosis, manifested clinically as tenderness, warmth, edema, and bluish discoloration. Echo-Doppler revealed catheter-related thrombophlebitis with left iliocaval vein occlusion. The catheter was immediately removed. Because the thrombus exceeded the renal veins, insertion of a cava filter was impossible. Because of the risk of intracranial bleeding, high-dose anticoagulant therapy was contraindicated. Surgical thrombectomy was performed and a white clot was located and removed. At the same time, a decrease in the platelet count made us suspect heparin-induced thrombocytopenia, caused by using heparinized saline as a flush to maintain the patency of the arterial catheter. As a result, the patient was treated with a low dose of danaparoid, which was replaced after three weeks by fluindione for six months. ELISA tests to detect antibodies against heparin and PF4 were finally negative.

### Case report 2

A 42-year-old French Caucasian man was admitted to our hospital with a 60% total body surface area full-thickness burn. Shortly after arrival, he required escharotomies (day 1). On day 2, the patient was taken to the operating room for the first staged excision of his burn wounds. A triple lumen Alsius Icy catheter (outer diameter at insertion site: 9.3 F, length: 38 cm) was placed in the inferior vena cava via the right femoral vein and active core warming of the patient was begun. The patient maintained a core body temperature within 1.5°C of his starting body temperature of 36.7°C during the entire surgery. He came back to the operating room on days 3 and 5 for dressing changes and on day 4 for a new burn excision. During this period, active core warming of the patient was maintained. On day 7, he presented with a biological inflammatory syndrome. Echo-doppler revealed a 4-cm-long thrombus at the femoral catheter site with complete blood flow obstruction. The catheter was immediately removed. Blood cultures and catheter tip were positive for methicillin-resistant S*taphylococcus aureus*. This septic thrombophlebitis was treated with catheter removal, parenteral antibiotics, and anticoagulation therapy. Computed tomography scan did not show pulmonary embolus. Transesophageal echocardiography did not show valvular vegetations or intracardiac thrombus. The patient required anticoagulation therapy for the next six months.

## Discussion

In theory, the risk for catheter-related thrombus formation (which is inherent in any indwelling device) could increase with cooling and warming catheters [1]. However, none of the studies performed thus far have reported an increased risk for thromboses [1]. Using clinical examination and routine ultrasound, Pichon and colleagues [4], did not diagnose any deep venous thromboses at the time of catheter removal from a series of 40 patients. In their study, mild induced hypothermia (MIH) was maintained for a mean of 37 ± 6 hours and catheters were withdrawn during the 24 hours following the end of MIH. Al-Senani and colleagues [5], did not describe any clinically evident femoral venous damage or thrombosis from catheters in a series of 13 patients; however, post-cooling leg vein ultrasounds were not obtained routinely. Furthermore, the catheter use period for this trial was not explicitly stated. To the best of our knowledge, the only incident of possible cooling catheter-related thrombosis was published by Taylor and colleagues [3]. In a series of 11 traumatized patients, one patient experienced a non-fatal pulmonary embolus; subsequent diagnostic workup revealed a deep vein thrombosis at the femoral catheter site. This event occurred one week after the rewarming catheter was removed and replaced over a guidewire with a standard triple-lumen central venous catheter. The patient was treated with anticoagulation without additional complications. Catheter-related thrombosis is a multicausal disease. The individual risk of thrombosis arising in a patient is a result of the interactions between patient characteristics (inherited and acquired risk factors) and the catheter [6]. With regard to the catheter, it does not appear that the flow-impedance effect of the catheter's slightly larger cross-sectional area (due to the balloons) contributes to the local intravascular formation of clots [3]. There is a need for investigation of the potential thrombogenic effects of the warming balloons on the surface of the intravascular portion of the catheter. Thus far, the incidence has not been shown to be different when the system is used for therapeutic cooling and subsequent rewarming of patients who sustained acute intracranial or cardiac events [3]. However, it remains to be clinically proven that warm (42°C) balloons do not increase the risk of surface clot formation [3]. Furthermore, the amount of time that a catheter stays in place is a major risk factor. The manufacturer currently recommends a maximum use period of 7 days with the CoolLine catheter and 4 days with the Icy catheter. Studies evaluating the thrombogenicity of the catheter showed no abnormalities after being left in place for 4 days [5]. No data are available regarding longer indwelling times [1]. The safety of prolonged use of this endovascular cooling system remains to be investigated, but one of the two case reports presented here may illustrate increased risk for thrombosis after 7 days. Furthermore, the CoolLine catheter, which contains only two cooling balloons, allows for longer indwelling times than does the Icy catheter with three cooling balloons, but with a probably lower capacity for temperature management. Septic thrombophlebitis is one of the most serious complications related to central venous catheter (CVC) use [6]. Central venous catheter-related thrombosis and central venous catheter-related infections have been reported to be associated [7]. Catheter-related thromboses can become infected with persistent bacteremia; however, it has also been recognized that CVC infection increases the risk of thrombosis [6]. Concerning individual patient characteristics, prothrombotic states may play an important role in the development of CVC-related thrombosis. Hereditary prothrombotic states of clinical importance include factor V Leiden; prothrombin mutation; protein C, protein S and antithrombin deficiency; sickle cell disease; and hyperhomocysteinemia. Major acquired prothrombotic states include cancer, myeloproliferative disorders, antiphospholipid syndrome, and heparin-induced thrombocytopenia. Anticipating the risk of CVC-related thrombosis and identifying high-risk patients who are prone to developing thrombosis is essential for initiating early preventive measurements such as prophylactic anticoagulation therapy.

## Conclusion

Although generally considered safe, cooling and warming catheters can be associated with mechanical complications such as catheter-related venous thrombosis. Intensivists who use these devices should be aware of this possible complication. In subsequent case-controlled studies, careful attention should be paid to the incidence of catheter-related infections or local venous thromboses. We recommend that systematic ultrasound of the veins be performed after catheter removal to exclude potential development of subclinical deep vein thrombosis. Finally, as with any other invasive catheter, to reduce the risk of complications, the catheter should be removed promptly when no longer needed. In all cases, the maximum use periods recommended by the manufacturer must be strictly followed.

## Abbreviations

CVC: central venous catheter; MIH: mild induced hypothermia.

## Consent

Written informed consent was obtained from the patient for publication of this case report. A copy of the written consent is available for review by the Editor-in-Chief of this journal.

## Competing interests

The authors declare that they have no competing interests.

## Authors' contributions

Each of the authors cared for at least one of the two patients. BP, GL and RP drafted the manuscript. JB and ED looked for the researches made before related with the manuscript. PG revised the manuscript. All authors read and approved the final manuscript.
